# Genetic Diversity and Population Structure of Tetraploid Wheats (*Triticum turgidum* L.) Estimated by SSR, DArT and Pedigree Data

**DOI:** 10.1371/journal.pone.0067280

**Published:** 2013-06-27

**Authors:** Giovanni Laidò, Giacomo Mangini, Francesca Taranto, Agata Gadaleta, Antonio Blanco, Luigi Cattivelli, Daniela Marone, Anna M. Mastrangelo, Roberto Papa, Pasquale De Vita

**Affiliations:** 1 Consiglio per la Ricerca e la sperimentazione in Agricoltura, Cereal Research Centre, Foggia, Italy; 2 Department of Soil, Plant, and Food Sciences, Section of Genetics and Plant Breeding, University of Bari, Via Amendola, Bari, Italy; University of Milano Bicocca, Italy

## Abstract

Levels of genetic diversity and population genetic structure of a collection of 230 accessions of seven tetraploid *Triticum turgidum* L. subspecies were investigated using six morphological, nine seed storage protein loci, 26 SSRs and 970 DArT markers. The genetic diversity of the morphological traits and seed storage proteins was always lower in the durum wheat compared to the wild and domesticated emmer. Using Bayesian clustering (K = 2), both of the sets of molecular markers distinguished the durum wheat cultivars from the other tetraploid subspecies, and two distinct subgroups were detected within the durum wheat subspecies, which is in agreement with their origin and year of release. The genetic diversity of morphological traits and seed storage proteins was always lower in the improved durum cultivars registered after 1990, than in the intermediate and older ones. This marked effect on diversity was not observed for molecular markers, where there was only a weak reduction. At K >2, the SSR markers showed a greater degree of resolution than for DArT, with their identification of a greater number of groups within each subspecies. Analysis of DArT marker differentiation between the wheat subspecies indicated outlier loci that are potentially linked to genes controlling some important agronomic traits. Among the 211 loci identified under selection, 109 markers were recently mapped, and some of these markers were clustered into specific regions on chromosome arms 2BL, 3BS and 4AL, where several genes/quantitative trait loci (QTLs) are involved in the domestication of tetraploid wheats, such as the tenacious glumes (*Tg*) and brittle rachis (*Br*) characteristics. On the basis of these results, it can be assumed that the population structure of the tetraploid wheat collection partially reflects the evolutionary history of *Triticum turgidum* L. subspecies and the genetic potential of landraces and wild accessions for the detection of unexplored alleles.

## Introduction

It has been widely reported that the genetic diversity of the elite germplasm for the major crops has suffered an overall reduction with time in comparison to the wild forms, especially for self-pollinating cereals such as durum wheat. This has arisen primarily as a consequence of the combined effects of domestication processes, recurrent use of adapted germplasm, and adoption of breeding schemes that do not favour wide genetic recombination [1]–[5]. Durum wheat (*Triticum turgidum* ssp. *durum*) is the only tetraploid species with significant agricultural importance, because of its use for human consumption (e.g., pasta, cous cous, bread, bulgur). Since modern varieties of durum wheat are expected to have very strict quality parameters (i.e., protein content, gluten quality, semolina colour), the use of wide crosses with unadapted germplasm in breeding programmes is very limited and selection intensity is very high. Through drift and selection, a large amount of genetic diversity has been lost, which has thus reduced the potential for wheat improvement in modern agricultural systems [6]–[9]. In this context, landraces, wild forms and other related wild species can have crucial roles in breeding programmes [10]–[12] because of their wide variability in terms of phenological, morphological, abiotic, biotic and quality traits. The evaluation of the level and structure of genetic diversity in tetraploid wheats is a prerequisite for plant breeding and genetic resource conservation programmes.

The level and structure of genetic diversity can be estimated by different approaches, which include the use of pedigrees [13], biochemical and morphological markers [14]–[15], and molecular markers [9], [16]–[18]. Pedigree analysis has been extensively used to define the genetic diversity in barley [17], [19], maize [20], rice [21], bread wheat [22] and durum wheat [23]–[24]. Unfortunately, the degree of genetic relatedness among genotypes based on pedigree information can sometimes be erroneous or incomplete, and it does not necessarily reflect the underlying genetics [23]. The accuracy of a coefficient of parentage depends on the availability of reliable and detailed pedigree data. In addition, these calculations do not take into account the effects of selection, mutation, and genetic drift, and require several simplifying assumptions that are generally not met.

In contrast, DNA markers allow the assessment of relatedness directly at the DNA level by estimation of the proportion of alleles that are identical by state, with the underlying assumption of a strict relationship between identity by state and identity by descent. In this context, the extent of the information that they can provide might depend on the nature and number of markers (e.g., level of homoplasy, mutation rate), the genome coverage and distribution, and the population under investigation [25], [16].

Different molecular markers can be used for genome analysis, and many of these have been applied with success for genetic mapping [26]–[27], phylogenetic relationships [28], comparative genomic studies, and diversity studies [9], [29]. Many studies have demonstrated the effectiveness of simple sequence repeat (SSR) markers for the characterisation of germplasm collections, because of their ease of use, high polymorphism, locus specificity, and codominance [9], [23], [30]. Diversity array technology (DArT) markers are also widely used for genome analysis; these offer deep genome coverage and high effectiveness, as many genotypes can be screened in a time-effective and cost-effective manner [9], [31]–[32]. More recently, the availability of the sequences of 2,000 wheat DArT clones (http://www.diversityarrays.com/sequences) has provided functional meaning to these markers, opening a number of applications, such as collinearity studies, fine mapping of loci of interest, and identification of candidate genes in association mapping [33].

Tetraploid wheats are genetically and morphologically diverse and their evolution under domestication has not been fully elucidated [34]–[35]. Almost all of the studies conducted to date have considered the subspecies of the tetraploid wheat (*Triticum turgidum* L.) separately for the analysis of genetic diversity. So far, ssp. *durum* [23]–[24], ssp. *dicoccum* [36]–[37], ssp. *polonicum* [38] and ssp. *dicoccoides* [39] have only rarely been analysed together [40]–[41]. In this context, the objectives of our study were to: (i) characterise the population structure and level of genetic diversity in a collection of 230 accessions of seven tetraploid *Triticum turgidum* L. subspecies using morphological traits, biochemical markers, SSR and DArT markers; (ii) assess the correspondence between similarity matrices based on these different types of traits; and (iii) consider the potential of the DArT markers to detect genomic regions that have been potentially subjected to selection events. The study was also focused on analysis of temporal diversity changes in durum wheat cultivars released during the recent breeding period.

## Materials and Methods

### Plant Material

The tetraploid wheat (*T. turgidum* L., 2 n = 4x = 28; AABB genome) collection was classified according to van Slageren [42] and MacKe [43], who considered all forms as subspecies of *T. turgidum* (Table 1). This consists of 230 accessions classified into seven subspecies: ssp. *durum* (128), ssp. *turanicum* (20), ssp. *turgidum* (19), ssp. *polonicum* (20), ssp. *carthlicum* (12), ssp. *dicoccum* (19) and ssp. *dicoccoides* (12).

**Table 1:** List of accessions of the *T. turgidum* subspecies included in the wheat collection.

Taxonomic classification	Accession	Country	Taxonomic classification	Accession	Country
*T. turgidum*	Cappelli	Italy	*T. turgidum*	K cer	Egypt
ssp. *durum*	Aziziah	Italy	ssp. *turanicum*	Cltr-11390	United State
	Russello	Italy		PI 68287	Azerbaijan
	Timilia	Italy, Sicily		PI 113393	Iraq
	Taganrog	Italy		PI 167481	Turkey, Denizli
	Capeiti-8	Italy		PI 191599	Marocco, Rabat-Sale
	Grifoni	Italy		PI 192641	Morocco
	Kyperounda	Marocco		PI 254206	Iran
	Langdon	United States		PI 278350	Italy
	Hymera	Italy		PI 290530	Hungary, Pest
	Trinakria	Italy		PI 306665	France, Herault
	Appulo	Italy		PI 576854	Turkey, Diyarbakir
	Belfuggito	Italy		PI 623656	Iran, West Azerbaijan
	Lambro	Italy		PI 624429	Iran, Bakhtaran
	Creso	Italy		PI 127106	Afghanistan, Faryab
	Isa	Italy		PI 67343	Australia, Victoria
	Mexicali 75	Mexico		PI 192658	Morocco
	Mida	Italy		PI 184526	Portugal
	Polesine	Italy		PI 352514	Azerbaijan
	Valgerardo	Italy		PI 362067	Romania, Brasov
	Valnova	Italy	*T. turgidum*	PI 56263	Portugal, Lisboa
	Tito	Italy	ssp. *turgidum*	PI 134946	Portugal, Lisboa
	Sansone	Italy		PI 157983	Italy, Sicily
	Karel	Italy		PI 157985	Italy, Sicily
	Produra	United States		PI 173503	Turkey, Artvin
	Valforte	Italy		PI 185723	Portugal, Leira
	Berillo	Italy		PI 191104	Spain
	Appio	Italy		PI 191145	Spain, Baleares
	Athena	Italy		PI 191203	Spain
	Latino	Italy		PI 286075	Poland
	Messapia	Italy		PI 221423	Portugal
	Arcangelo	Italy		PI 352544	Switzerland, Vaud
	Lloyd	United States		PI 290522	Germany
	Altar84	Mexico		PI 290526	Hungary, Pest
	Duilio	Italy		PI 341391	Turkey, Burdur
	Primadur	France		PI 352538	United Kingdom
	Quadruro	Italy		PI 352541	Germany
	Tresor	Italy		PI 352542	France
	Adamello	Italy		PI 352543	France
	Grazia	Italy	*T. turgidum*	PI 266846	United Kingdom, England
	Ambral	France	ssp. *polonicum*	PI 278647	United Kingdom, England
	Amedeo	Italy		PI 289606	United Kingdom, England
	Brindur	France		PI 330554	United Kingdom, England
	Neodur	France		PI 330555	United Kingdom, England
	Agridur	France		PI 349051	Georgia
	Antas	Italy		PI 352487	Germany, Saxony-Anhalt
	Plinio	Italy		PI 352488	Italy
	Simeto	Italy		PI 352489	Cyprus
	Fenix	Italy		PI 361757	Denmark
	Ofanto	Italy		PI 366117	Egypt, Sinai
	Enduro	Italy		PI 387479	Ethiopia
	Cirillo	Italy		PI 566593	United States
	Cosmodur	France		PI 208911	Iraq
	Dauno	Italy		PI 210845	Iran
	Doral	France		PI 223171	Jordan
	Exeldur	France		PI 272564	Hungary, Pest
	Fauno	Italy		PI 286547	Ecuador
	Gianni	Italy		PI 290512	Portugal
	Granizo	Spain		PI 306549	Romania
	Parsifal	France	*T. turgidum*	Citr 7665	Russian Federation
	Zenit	Italy	ssp. *carthlicum*	PI 70738	Iraq
	Italo	Italy		PI 94755	Georgia
	Kronos	United States		PI 115816	Georgia
	Ceedur	France		PI 283888	Iran
	Arcobaleno	Italy/Spain		PI 341800	Russian Federation, Dagest
	Ares	Italy		PI 499972	Georgia
	Colosseo	Italy		PI 532501	Former Soviet Union
	Fortore	Italy		PI 572849	Georgia
	Platani	Italy		PI 573182	Turkey, Kars
	Preco	Italy		PI 585017	Georgia
	Saadi	France		PI 585018	Georgia
	Bronte	Italy	*T. turgidum*	Farvento	Italy
	Ciccio	Italy	ssp. *dicoccum*	Lucanica	Italy
	Durfort	France		Molise selezione Colli	Italy
	Iride	Italy		ISC Foggia 152	Iran
	Nefer	France		ISC Foggia 159	Morocco
	Rusticano	Italy		ISC Foggia 161	United Kingdom
	San Carlo	Italy		ISC Foggia 171	Ethiopia
	Svevo	Italy		ISC Foggia 175	Hungary
	Vitromax	Italy/Spain		MG 5350	Ethiopia
	Varano	Italy		MG 4387	United Kingdom
	AC-Navigator	Canada		MG 5416/1	Iran
	Baio	Italy		MG 5471/1	Spain
	Cannizzo	Italy		MG 5473	Spain
	Claudio	Italy		MG 15516/1	Syria
	Martino	Italy		MG 5344/1	Ethiopia
	Provenzal	Italy		MG 5293/1	Italy
	Giotto	Italy		MG 5323	n.a.
	Meridiano	Italy		MG 3521	n.a.
	Orobel	Italy		MG 5300/1	n.a.
	Quadrato	Italy	*T. turgidum*	PI 346783	Hungary, Pest
	Vesuvio	Italy	ssp. *dicoccoides*	PI 343446	Israel
	Avispa	Italy		PI 481539	Israel
	Fiore	Italy		PI 352323	Asia Minor
	Tiziana	Italy		PI 352324	Lebanon
	Duetto	Italy		PI 355459	Armenia
	Dylan	Italy		PI 470944	Syria, Al Qunaytirah
	Grecale	Italy		PI 470945	Syria, Al Qunaytirah
	Normanno	Italy		MG 4343	n.a.
	Virgilio	France		MG 4328/61	n.a.
	Ancomarzio	Italy		MG 5444/235	n.a.
	Casanova	Italy		MG 4330/66	n.a.
	Chiara	Italy			
	Latinur	France			
	Vendetta	Italy			
	L092	United States			
	L252	United States			
	Maestrale	Italy			
	Orfeo	Italy			
	S99B34	United States			
	Saragolla	Italy			
	Ariosto	Italy			
	Arnacoris	Italy			
	Canyon	Italy			
	Imhotep	Italy			
	PR22D89	Italy			
	Strongfield	Canada			
	Alemanno	Italy			
	Ciclope	Italy			
	K26	Italy			
	UC1113	Canada			
	Neolatino	Italy			
	5-BIL42	Italy			
	PC32	Italy			
	Barcarol	Italy			
	Pedroso	Spain			
	Sharm 5	Syria			
	West Bread 881	United States			
					

n.a. not available. CItr and PI number indicate the accession number in USDA National Small Grains Collection, Aberdeen, Idaho, USA. MG number indicate the accession number in CNR Institute of Plants Genetics, Bari, Italy. ISC Foggia number indicate the accession number in CRA-CER Cereal Research Centre, Foggia, Italy.

The durum wheat accessions (96 are mainly elite cultivar) are representative of the Italian durum breeding programmes over the last 100 years. A further subset of these accessions (32) came from the most important durum production areas (Table 1). For the purpose of the study, the durum collection was subdivided into three groups according to the year in which each cultivar was released. Group 1 comprises the ‘old’ genotypes that were selected from indigenous and exotic landraces, and/or varieties selected from crosses involving landraces (from 1915 to 1973); group 2 comprises the ‘intermediate’ genotypes that were selected from crosses between CIMMYT breeding lines and group 1 materials (from 1974 to 1989); and group 3 comprises the ‘modern’ genotypes selected after the 1990s. Twenty plants of each accession were sown at Valenzano (Bari, Italy), and a single plant representing the prevalent biotype of the accession was selected and grown to maturity for self-seeding. Seed stocks can be obtained upon request from the corresponding author.

### Morphological and Biochemical Analysis

The accessions were evaluated according to six morphological traits recorded following the descriptors defined by the International Plant Genetic Resources Institute (http://www.cgiar.org/ipgri). In particular, during the growing season of 2008–2009, outer glume colour, awn colour, awned/awnless form, and spike and culm glaucousness were recorded, while the trait of naked/hulled kernel was evaluated at harvest time. The collection was investigated for glutenin alleles located at five loci: *Glu-A1* and *Glu-B1* for the high-molecular-weight subunits (HMW-GS), and *Glu-A3, Glu-B3* and *Glu-B2* for the low-molecular-weight subunits (LMW-GS). The glutenins were extracted from flour samples (50 mg) according to Laemmli [44]. Electrophoresis was performed in an SE 600 Ruby Hoefer vertical electrophoresis unit with stacking and running gel concentrations of 3% and 10% acrylamide, respectively. The identification of HMW-GS was based on the classification of Branlard *et al.* [45]. The new subunits and alleles were designed according to Li *et al.* [46], McIntosh *et al.* [47] and Riefolo *et al.* [14]. The LMW-GS were classified according to Nieto-Taladriz *et al.* [48]. Moreover, the collection was assessed for gliadin alleles at four loci: *Gli-A1, Gli-B1, Gli-A2* and *Gli-B2*. Gliadins were extracted from single seeds and fractioned using acid polyacrylamide gel electrophoresis (pH 3.1), according to Lafiandra and Kasarda [49], with their classification according to Riefolo *et al.* [14], Boggini *et al.* [50] and Aguiriano *et al.* [51].

### DNA Extraction and SSR and DArT Analyses

Leaf tissue of the plants that represented the prevalent biotype of each accession was used for DNA extraction, using the protocol described by Sharp *et al.* [52]. The wheat collection was genotyped with 26 SSR markers, which were selected based on published map data [53]–[55] according to the following criteria: locus-specific amplification, low complexity, robust amplification, and good genome coverage (nearly one marker per chromosome arm). PCR amplification was carried out in 15 µl volumes containing 2 µl DNA (≈ 80 ng), 1.5 µl 10× PCR buffer (EuroClone), 0.4 µM of each microsatellite primer (the forward primers were fluorescently labelled), 1.5 mM MgCl_2_ (EuroClone), 0.2 mM dNTP mixture (Fermentas), and 1 U Taq DNA-polymerase (EuroClone). The PCR was carried out as follows: 95°C for 3 min, followed by 35 cycles of 94°C for 30 s, the specific T_a_ for each primer for 30 s, 72°C for 1 min, with a final extension at 72°C for 10 min. The PCR products were detected by capillary electrophoresis using an ABI PRISM 3130xl analyser, and analysed using GeneMapper version 4.0 genotyping software. The internal molecular-weight standard was 500-ROX (Life Technologies).

Genotyping with DArT markers was performed by Triticarte Pty. Ltd. (Canberra, Australia; http://www.triticarte.com.au), a whole-genome profiling service laboratory, as described by Akbari *et al.* [31]. For both the SSR and DArT markers, alleles that occurred at a low frequency (p<0.05) were excluded from the analysis.

### Co-ancestry Analysis

The pedigree records, as reported in Table S1, were obtained from the literature, web-based pedigree databases (http://genbank.vurv.cz/wheat/pedigree), and personal communication with the breeders. For some of the genotypes, the pedigree data were not available and/or consistent, and therefore a subset of 116 varieties for which the pedigree data could be traced back several generations was defined (see Table S1). The coefficient of co-ancestry between the genotypes, or the ‘Kinship coefficient’, was calculated using Winkin2, according to Tinker and Mather [56]. The coefficient of co-ancestry was assumed to be zero (f = 0) in the absence of any degree of kinship, and one (f = 1) when the maximum degree of kinship was observed. A kinship coefficient matrix was obtained and transformed into a distance matrix (using d = 1 - r), which was used for the multivariate analysis.

Mantel test genetic similarity (GS) matrices for the SSR and DArT markers, and the distance matrix obtained from the kinship values, were used for pair-wise comparisons to determine the degrees of association between pairs of matrices, using the ARLEQUIN software, version 3.5 [57]. The normalised Mantel statistic Z [58] was used to determine the level of association between the matrices. The association was considered significant if r ≥0.50, p<0.01.

### Genetic Diversity, Genetic Structure and Population Differentiation Analysis

The genetic diversity (morphological traits, seed storage protein loci, and molecular markers, separately) within each subspecies was estimated by calculation of the number of observed alleles (na) and the unbiased estimator of gene diversity (H_E_ [59]) as:
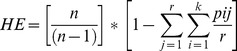
where *pij* is the frequency of the *i* ^th^ variants at the *j* ^th^ locus, and *r* is the number of loci.

The population structure was determined on 230 tetraploid wheat accessions. The molecular data (26 SSR and 970 DArT markers) were processed using the STRUCTURE program, version 2.2 (http://pritch.bsd.uchicago.edu/structure.html) [60]. The number of sub-groups (K) was estimated by 20 independent runs for each K (from 2 to 20) applying the admixture model, with allele frequencies uncorrelated for SSR markers and correlated for DArT markers, 100,000 Markov Chain Monte Carlo (MCMC) repetitions, and a 100,000 burn-in period. The means of the log-likelihood estimates for each K were calculated. The true K was determined using both an estimate of the posterior probability of the data for a given K (as proposed by Pritchard *et al.*, [60]), and the Evanno ΔK [61]. Successively, separate analyses for the genetic diversity structure were performed on each sub-group, with the same parameters previously set. A genotype was considered to belong to a group if its membership coefficient was ≥0.50 [30]. For the population structure, we used the Spearman’s rank correlation coefficient (ρ), to correlate the year of release of the durum cultivars and the taxonomic classification of the genetic structure. Dendrograms constructed with the neighbour-joining (NJ) algorithm from shared-allele distances were also used to analyse the genetic structure of the collection, which was implemented in the PAST software, version 2.1 [62].

The population differentiation was assessed by analysis of molecular variance (AMOVA) using the ARLEQUIN software, version 3.5 [57]. The significance levels for the variance components were estimated using 16,000 permutations. Loci under selection were identified by the *Fst*-outlier detection method, using 100,000 simulations. Based on the *Fst* values that fell outside the 99% confidence interval, candidate loci identified under positive selection were used for further analysis.

## Results

### Genetic Diversity for Morphological and Biochemical Traits

The wheat collection was evaluated for six morphological traits and nine seed-storage-protein loci (Table 2). Together with ssp. *turgidum*, ssp. *dicoccum* and ssp. *dicoccoides*, the ssp. *durum* showed a higher allele number than detected in ssp. *turanicum*, ssp. *polonicum* and ssp. *carthlicum*. The H_E_ of ssp. *durum* was the lowest (0.23), while ssp. *dicoccoides* showed the highest H_E_ (0.55).

**Table 2:** Alleles number and genetic diversity of morphological traits and seed protein storage loci for each subspecies included in the wheat collection.

	*T. turgidum*						
	ssp. *durum*	ssp. *turanicum*	ssp. *polonicum*.	ssp. *turgidum*	ssp. *carthlicum*	ssp. *dicoccum*	ssp. *dicoccoides*
Sample size	128	20	20	19	12	18	12
Glume colour	3	1	1	3	3	2	2
Awn colour	3	3	3	2	3	2	3
Glaucosness culm	2	2	1	1	1	1	2
Glaucosness spike	2	2	1	2	2	2	2
Awnedness	1	1	1	1	1	1	1
Naked/hulled kernel	1	1	1	1	1	1	1
n_a_ morphological traits	12	10	8	10	11	9	11
H_E_ morphological traits	0.09	0.15	0.07	0.23	0.28	0.07	0.33
*Glu-A1*	4	1	4	6	1	4	4
*Glu-B1*	10	5	2	7	4	11	5
*Glu-B2*	2	2	2	2	2	2	2
*Glu-A3*	3	3	4	4	2	5	3
*Glu-B3*	2	2	2	3	1	4	3
n_a_ *Glu-*	21	13	14	22	10	26	17
H_E_ *Glu-*	0.22	0.43	0.55	0.67	0.32	0.73	0.67
*Gli-A1*	4	1	2	2	2	2	5
*Gli-B1*	21	6	7	13	7	9	9
*Gli-A2*	3	2	2	2	2	2	2
*Gli-B2*	3	2	1	2	1	2	2
n_a_ *Gli-*	31	11	12	19	12	15	18
H_E_ *Gli-*	0.39	0.42	0.42	0.44	0.44	0.54	0.65
n_a_ total	64	34	34	51	33	50	46
H_E_ mean	0.23	0.33	0.35	0.45	0.35	0.45	0.55

*Glu-* glutenin locus; *Gli-* gliadin locus; n_a_: number of alleles; H_E_: genetic diversity.

The number of states of morphological traits ranged from 8 to 12. As expected, the awed/awned-less and naked/hulled kernel traits were monomorphic in each *T. turgidum* subspecies. The variability of the durum cultivars (H_E_ = 0.09) was lower than the ssp. *dicoccoides* accessions (H_E_ = 0.33).

The greatest allele number of glutenin loci was found in ssp. *dicoccum* (26 alleles), with the lowest in ssp. *carthlicum* (10 alleles). Three out of five glutenin loci analysed were polymorphic in each subspecies, with the exception of *Glu-A1*, monomorphic in ssp. *turanicum* and ssp. *carthlicum*, and *Glu-B2* monomorphic in ssp. *carthlicum*. *Glu-B1* was the most polymorphic locus (2–11 alleles). The H_E_s in the durum cultivars were low in comparison to the ssp. *dicoccum* and ssp. *dicoccoides*.

For gliadins, the allele number of loci ranged from 11 to 31. *Gli-A1* was monomorphic in ssp. *turanicum*, as well as the *Gli-B2* locus in ssp. *polonicum* and ssp. *carthlicum*. *Gli-B1* was the most polymorphic one (6–21 alleles).

### Genetic Diversity for SSR and DArT Markers

Twenty-six SSR loci broadly distributed over the genome and 970 DArT markers were used. The chromosomal position and number of alleles detected for each SSR are detailed in Table S2. A total of 436 alleles were detected, which ranged from 133 (ssp. *carthlicum*) to 211 (ssp. *durum*), while the number of alleles per locus varied from one (*BQ170801* and *BJ274952* for ssp. *carthlicum*) to 18 (*Xwmc606* for ssp. *durum*), with a mean of 16.8 alleles per locus. All of the genomic (g)SSRs were polymorphic in the seven subspecies, while the EST-SSRs were monomorphic in ssp. *carthlicum*. The largest allele number was detected for *Xwmc606* (6–18 alleles), while the lowest was found for *BJ274952* (1–3 alleles).

When considering the genetic diversity (H_E_) among the subspecies, the differences were less evident (Table 3). Indeed, using SSRs, the H_E_ values ranged from 0.56 (ssp. *carthlicum*) to 0.70 (ssp. *dicoccoides*), and when computed for the DArT markers, from 0.22 (ssp. *carthlicum*) to 0.33 (ssp. *durum*), with a mean value of 0.28.

**Table 3:** Genetic diversity for SSR and DArT markers for each subspecies included in the wheat collection.

		*T. turgidum*							
		ssp. *durum*	ssp. *turanicum*	ssp. *polonicum*	ssp. *turgidum*	ssp. *carthlicum*		ssp. *dicoccum*	ssp. *dicoccoides*
	Sample size	128	20	20	19	12	18		12
								
SSRs	H_E_ mean	0.60	0.58	0.58	0.62	0.56	0.66		0.70
								
DArTs	H_E_ mean	0.33	0.29	0.28	0.28	0.22	0.29		0.30

### Population Structure of the Wheat Collection

**Figure 1:**
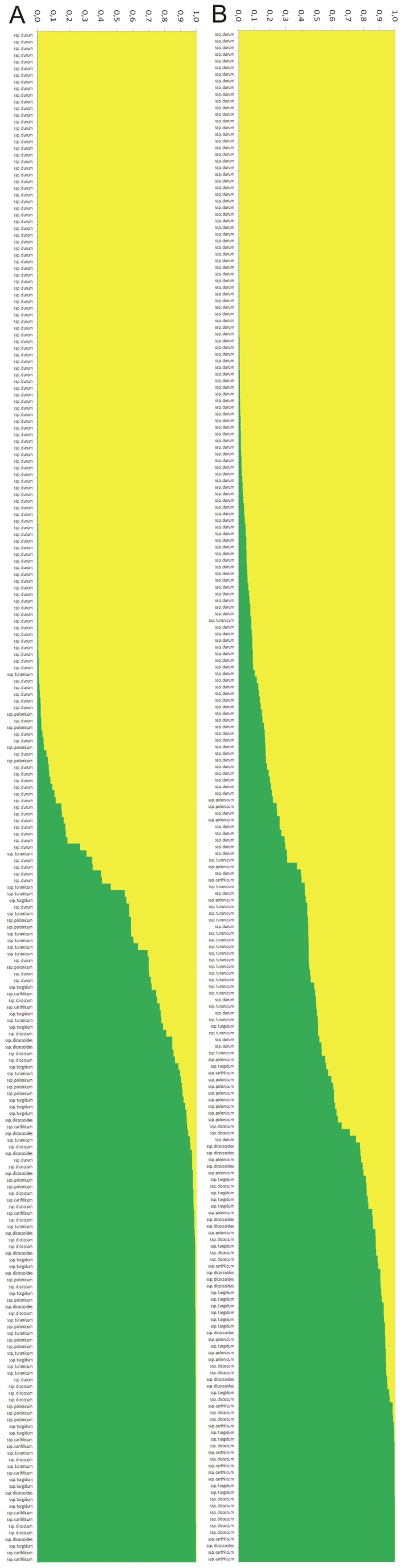
Population structure of the 230 accessions using SSR and DArT markers, as estimated using the model-based Bayesian algorithm implemented in the STRUCTURE programme (K = 2).

The population structure was analysed using a Bayesian approach on 230 wheat accessions implemented in the STRUCTURE software. Following the method of Evanno *et al.* [61], the ΔK were plotted against the K numbers of the sub-groups. The maximum ΔK occurred at K = 2 and K = 7 for the SSR markers, and at K = 2 and K = 3 for the DArT markers. When considering K = 2, the collection was split in two sub-groups (group 1, group 2) containing 129 and 101 accessions based on SSRs data, respectively, and 147 and 83 accessions based on DArT data, respectively (Figure 1). In both cases, the structures assigned all of the durum accessions to the same group (group 1), with the exception of four cultivars (Timilia, Belfuggito, Lambro, Russello). Furthermore, based on the analysis carried out with only SSRs, Ceedur and Kyperounda were assigned to group 2, together with all of the other subspecies. In particular, all of the durum genotypes were assigned to cluster 1, with a q1 mean membership of 0.96 and 0.88 for SSRs and DArT, respectively. The remaining genotypes were assigned to group 2, with a q2 mean of 0.91 and 0.85 for SSRs and DArT, respectively, with the exception of some genotypes that showed high levels of admixture. Again, using DArTs, 31 genotypes showed q values lower than 0.6, while with SSRs, q values lower than 0.6 were only recorded for 10 genotypes. The difference in the sizes of these two groups was because when using DArT markers, most of the ssp. *turanicum* accessions (17 of 20) were assigned to the same group of ssp. *durum* with a greater number of admixed genotypes. Performing the analysis using DArT, at K = 3, the ssp. *turanicum* accessions were separated from the ssp. *durum*, while the remaining accessions were grouped together (data not shown).

**Figure 2:**
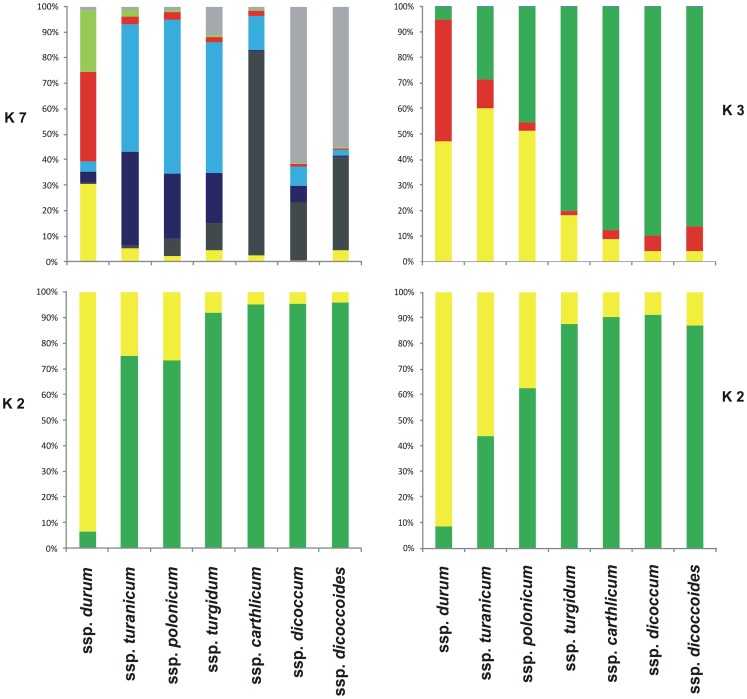
Population structure of each subspecies of wheat according to the taxonomical classification, using 26 SSR and 970 DArT markers.

Considering the mean q for each subspecies, at K = 2 (Figure 2), the data show the stratification for both of the sets of molecular markers. In particular, both sets distinguished the ssp. *durum* from the other subspecies, while the structure of the ssp. *turgidum* was relatively similar to other subspecies, such as ssp. *carthlicum*, ssp. *dicoccum* and ssp. *dicoccoides*. At K >2, the SSR markers showed a greater degree of resolution than the DArT, with their identification of a greater number of groups within each subspecies. In this case, with the exception of ssp. *durum*, the structure of ssp. *turgidum*, ssp. *turanicum*, and ssp. *polonicum* appeared to be more homogeneous and recognisable from other subspecies. At the same time, at K = 7, ssp. *carthlicum* showed a genetic structure significantly different from the other subspecies analysed, appearing more similar to ssp. *dicoccum* and ssp. *dicoccoides* using the DArT markers (K = 3).

**Figure 3:**
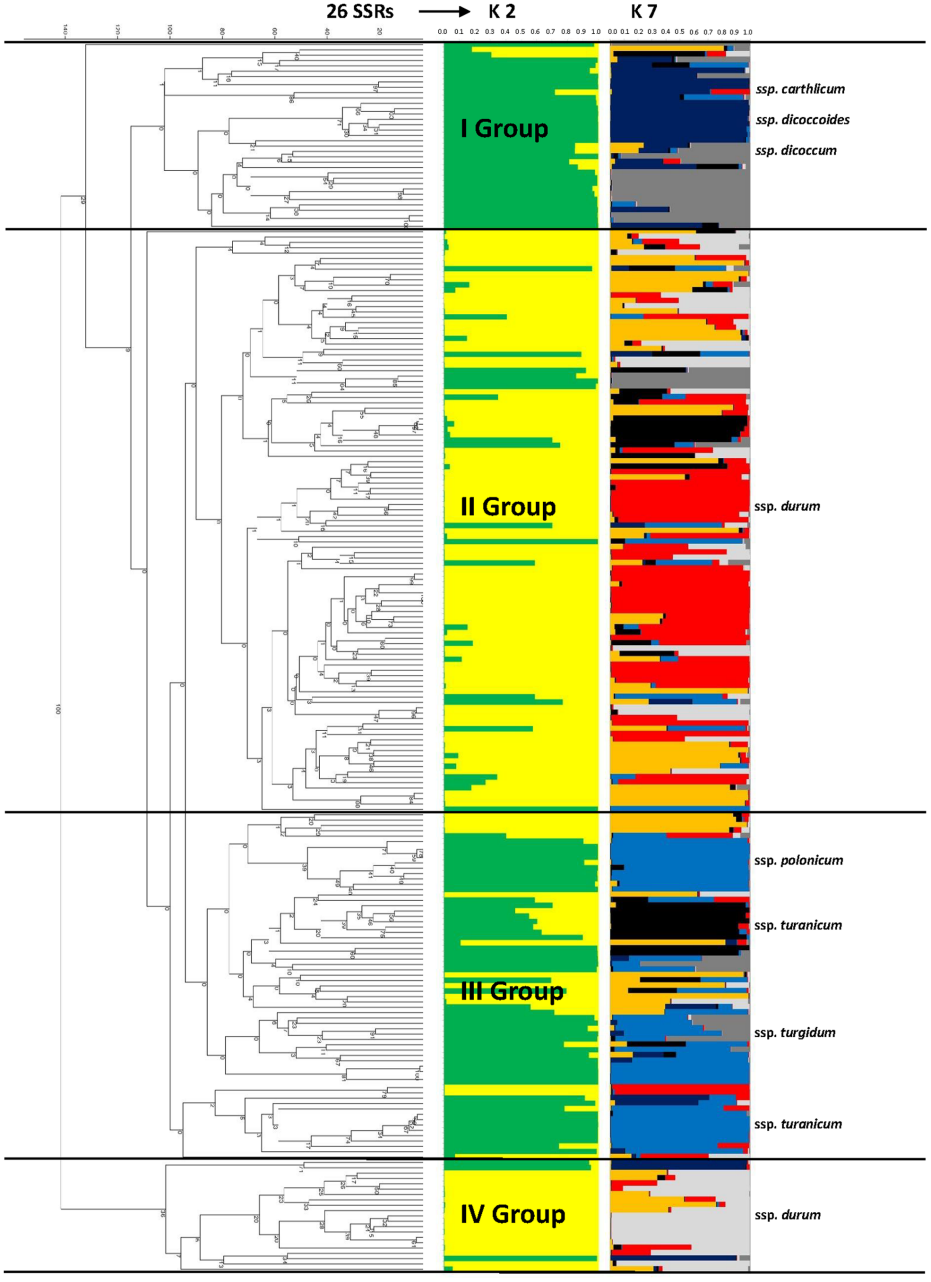
Population structure and dendrogram of the 230 accessions using SSR markers. The numbers on nodes are bootstrap probabilities estimated by permutation tests with 1000 replications.

**Figure 4:**
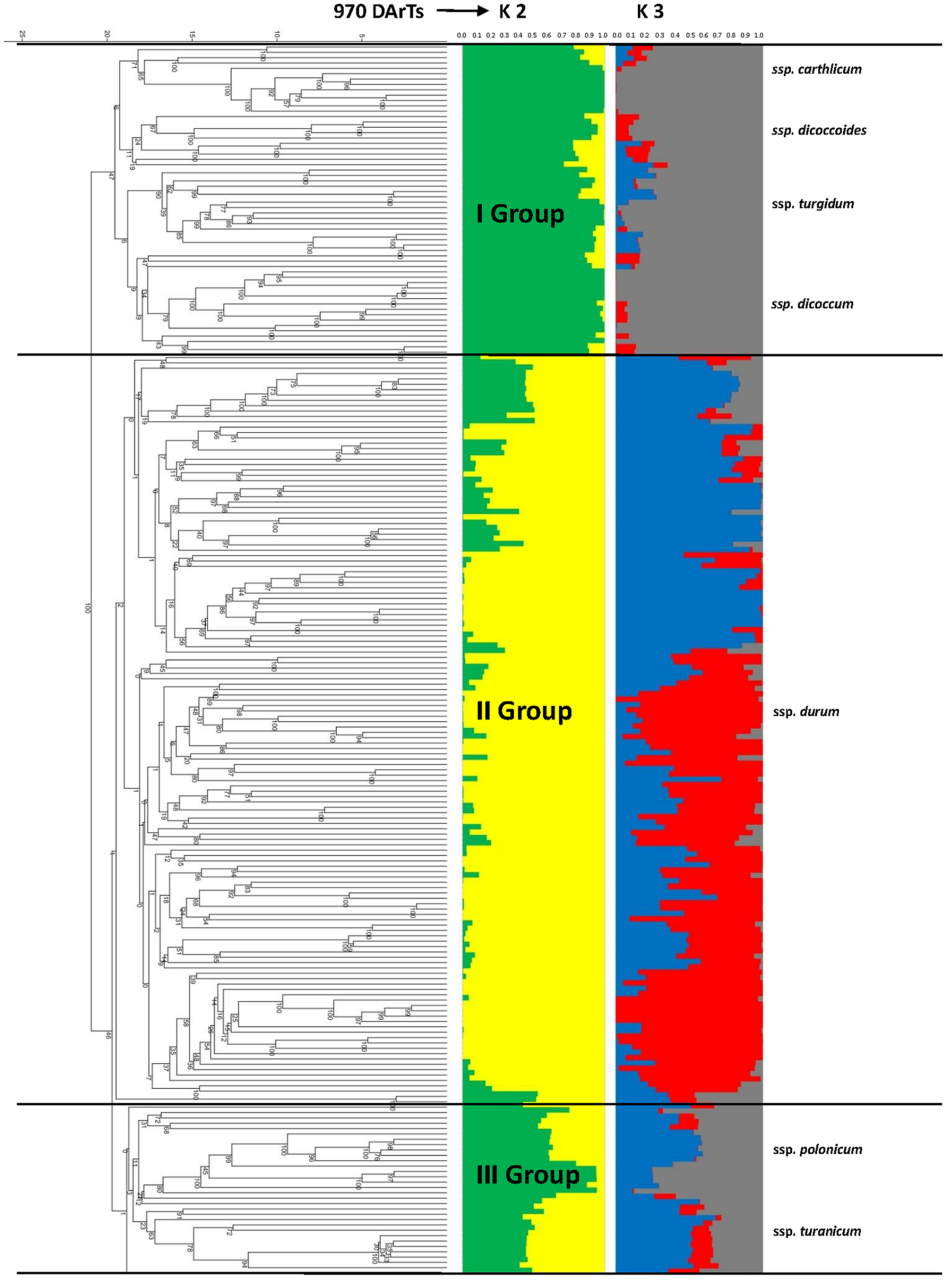
Population structure and dendrogram of the 230 accessions using DArT markers. The numbers on nodes are bootstrap probabilities estimated by permutation tests with 1000 replications.

To further refine the relationships between the tetraploid wheat accessions, cluster analyses were performed for each of the two marker types, and the general topology of these trees was compared with the Bayesian model structure shown in Figures 3 and 4. The genotypes were clustered into four and three large groups, corresponding to their botanical classification for the SSR and DArT markers, respectively. In particular, the dendrogram generated using the SSR matrices of similarity (Figure 3) assembled the ssp. *durum* genotypes into two main groups (groups II and IV) with 93 and 17 varieties, respectively, although a number of genotypes were admixed. The remaining accessions were clustered in the other two groups (groups I and III). The greater part of the accessions of ssp. *dicoccum* (14), ssp. *carthlicum* (10) and ssp. *dicoccoides* (8) belonged to group I, while group III comprised mainly the accessions of ssp. *polonicum* (13), ssp. *turanicum* (15) and ssp. *turgidum* (16), and some admixed accessions of ssp. *durum*, which were generally of unknown origin (i.e., Ceedur) or were derived from introgression with wild and domesticated wheat (Lambro and Belfuggito). Similar data are reported in Figure 4 using the similarity matrix generated by the DArT markers. In this case, three distinct groups were identified, although with lower mean genetic distances. The accessions of ssp. *dicoccum* (17), ssp. *dicoccoides* (12), ssp. *turgidum* (17), ssp. *carthlicum* (10) and 2 admixed accessions of ssp. *polonicum* were in group I. All ssp. *durum* accessions were in group II, together with 8 accessions of ssp. *turanicum*, 5 of ssp. *polonicum*, and 1 of ssp. *turgidum*. The largest part of ssp. *turanicum* (11) were in group III, together with a few accessions of ssp. *carthlicum* (2), ssp. *dicoccum* (2) and ssp. *turgidum* (1), and three old Italian durum varieties (Aziziah, Russello, Timilia).

To explore the relationships revealed using different types of data, the similarities between distance matrices based on the SSR, DArT, morphological and biochemical traits were measured using pair-wise comparisons among the genetic distance matrices with normalised Mantel statistics. The highest correlation was observed between the SSR and DArT matrices (r = 0.66, p≤0.001), which indicates that each set of markers yielded highly similar estimates of genetic distances between genotypes. The lowest values of correlation were recorded between morphological and biochemical matrices (r = 0.22, p≤0.001) and between morphological and DArT matrices (r = 0.28, p≤0.001).

### Divergence Analysis Among the Wheat Subspecies

The effects of selection pressure on the evolution of the cultivated forms of tetraploid wheat were determined by the fixation index (Fst). From comparisons of the alleles among genotypes of the seven subspecies and measurements of the level of differentiation for each DArT marker, the genetic changes selected over the course of the wheat breeding were assessed. Furthermore, knowing the nucleotide sequences of most DArT markers, it is possible to suggest candidate genes for the loci that are under positive selection between subspecies. On a total of 970 DArT markers that were polymorphic across the tetraploid collection, a subset of 590 DArT markers were positioned on a consensus map developed in durum wheat by Marone *et al*. [33].

The analysis of Fst on a locus-by-locus basis provided no statistical cut-off for the identification of loci that might be under positive selection. Therefore, we used an outlier detection method implemented in the ARLEQUIN software. Across the subspecies, a total of 211 outlier loci were identified as under positive selection, and of these, 109 (51.7%) were mapped in the consensus map [54].

**Figure 5:**
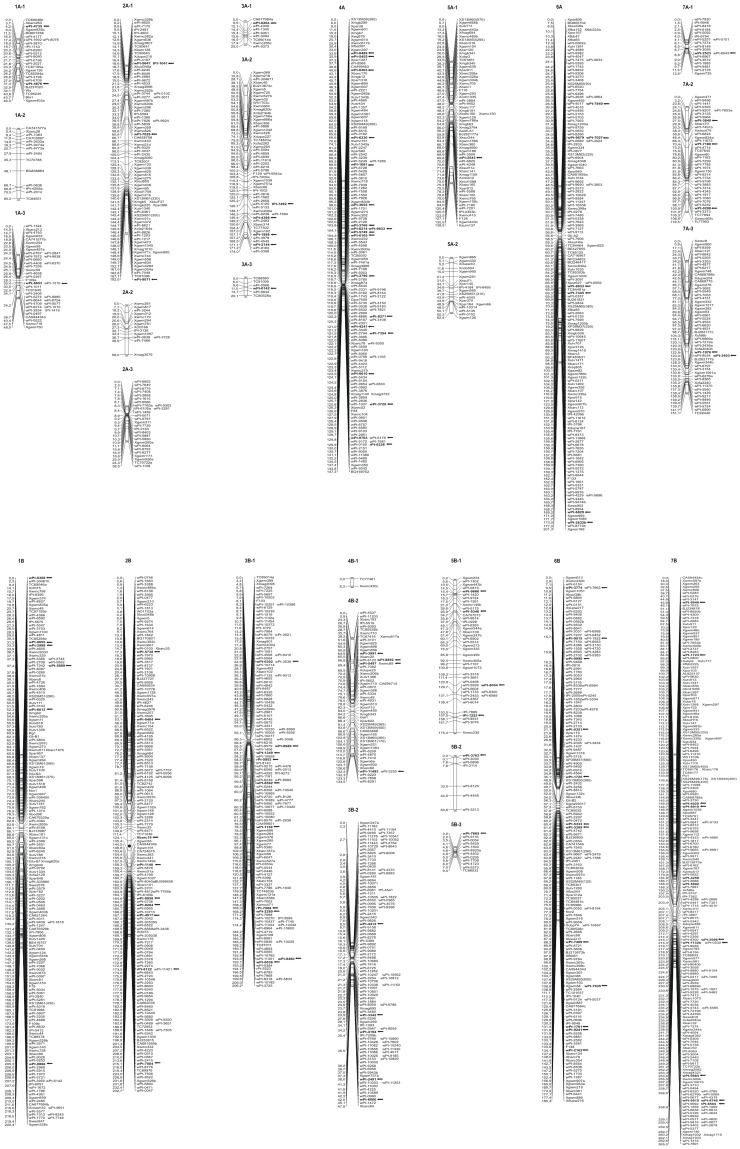
DArT markers under selection positioned (bold type and arrow) on a durum wheat consensus map (Marone et al. 2012a).

The markers under positive selection were spread across all chromosomes, with 42% and 58% of the markers located on genomes A and B, respectively (Table S3). In some cases, the markers under positive selection were spread over large chromosome regions, at great distances from each other, although regions in which a number of outlier markers were grouped together in a few cM were also identified on chromosomes 1B, 2B, 3B, 4A, 4B, 6A, 6B and 7B (Figure 5). Particularly interesting was the case of chromosome 4A, where three regions putatively under selection were found: 33.9–40.9 cM (three DArT markers), 89.9–104.2 cM and 118.5–129.6 cM (7 DArT markers). Among the 211 loci, NBS-LRR, protein kinase, peroxidase, putative cellulose synthase, and transposable element related sequences were identified as under selection between subspecies. These genes might be selected as having an important role in plant responses to biotic and abiotic stress, or, more simply, are in genetic linkage with the locus subjected to selection during the domestication process.

### Genetic Distance and Relationships between Durum Cultivars

**Figure 6:**
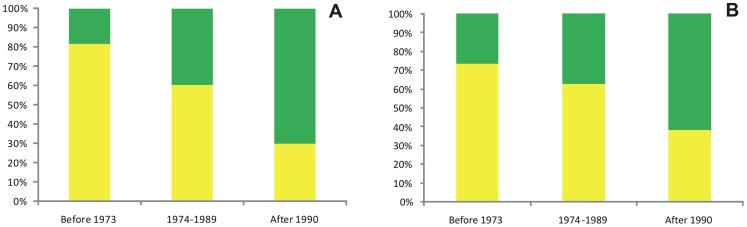
Population structure of the sub-group of durum cultivars inside the wheat collection with K = 2, using 26 SSR (A) and 970 DArT markers (B). The population structure of durum cultivars was grouped according to year of release.

The relationships between the durum cultivars were first established by analysing the genetic structure of 128 genotypes (Figure 6). The analysis carried out with both sets of markers (SSR and DArT) indicated that the maximum ΔK occurred at K = 2. The durum cultivars were split in two sub-groups according to their origin and year of release (Table S1). Indeed, a strong correlation was observed between the structure results (the q1 mean) and durum cultivars according to year of release (r = 0.39, 0.38, p≤0.001, using SSRs and DArT, respectively). The first sub-group included most of the historic Italian varieties that were largely established during the first three quarters of the last century, while the second sub-group was primarily represented by modern cultivars released after 1990, with some exceptions.

The temporal trend of diversity in the Italian durum collection is reported in Table 4, and to avoid the strong bias for different sampling sizes, we analysed the means of the genetic variation (H_E_) while combining the old and intermediate breeding groups in comparison with modern cultivars. One hundred and twenty-two out of 128 durum cultivars were grouped at K = 2 (SSR): 49 and 73 genotypes for the group before 1989 (old and intermediate) and after 1990 (modern), respectively. The number of alleles detected across the two groups were relatively similar for all of the traits, with the exception of the SSR markers, for which a similar level of diversity was found among the different groups (Table 4).

**Table 4:** Alleles number and genetic diversity across the two genetic groups of *T. turgidum* ssp. *durum* grouped according to years of release.

*T. turgidum*	Years of release	Sample size	Morphological traits		Glutenin protein		Gliadin protein		SSR markers	DArT markers	
			n_a_	H_E_	n_a_	H_E_	n_a_	H_E_	H_E_	H_E_	
ssp. *durum*	Old and intermediate	49	11	0.19	15	0.29	19	0.48	0.59		0.33
	Modern	73	10	0.07	17	0.19	17	0.34	0.60	0.32	

The differentiation of the two groups was also confirmed by the analysis carried out to estimate the presence of private alleles in each group. Considering a threshold of 5%, to eliminate the rare alleles, 3 SSR markers (Xgwm408, Xgwm537, Xgwm299) were polymorphic in the first group of old and intermediate durum wheat varieties, and monomorphic in the second group, while 5 markers, as 4 SSR (Xgwm1084, Xgwm1093, Xgwm1017, Xwmc606) and 1 DArT (wPt-4142), were polymorphic in the modern durum wheat group, and monomorphic in the first group released before 1989.

Using morphological and biochemical traits (glutenin and gliadin), there was a loss of genetic diversity (H_E_). Indeed, the ‘old and intermediate’ group showed H_E_ = 0.19, 0.29 and 0.48 for morphological, glutenin and gliadin traits, respectively, while the modern genotypes showed lower H_E_, at 0.07, 0.19 and 0.34 for the same traits, respectively. For the SSR markers, a two-fold higher mean H_E_ was recorded with respect to those of the morphological and storage protein, and no trend was observed between the two groups (H_E_ = 0.59, 0.60, respectively). The same behaviour was seen for DArT, with the H_E_ mean for the old and intermediate groups (0.33) higher that for the modern (0.32) cultivars. The data obtained with the SSR and DArT marker technologies were in agreement.

A subset of 116 durum cultivars selected on the basis of the pedigree information available was used to investigate the relationships between the genetic distances estimated with the SSR, DArT and pedigree data. For the pedigree data, a matrix of genetic distances was derived from the kinship coefficients established on the basis of the pedigree for each genotype. The genetic distances ranged from 0.04 to 0.96 for SSRs, from 0.003 to 0.49 for DArT, and from 0.0 to 1.0 for pedigree, with means of 0.64, 0.33 and 0.89, respectively. The lowest genetic distance (0.003) was observed between three pairs of genotypes by DArT markers (Dylan vs. Fenix, Latino vs. Maestrale, Belfuggito vs. Lambro), while Colosseo and Doral showed the lowest genetic distance (0.04) using the SSR markers. The highest genetic distances (1.0) were observed in the pedigree matrix between different pairs of genotypes with no shared ancestors, and they skewed the distribution towards higher values. The majority of the kinship coefficients were between 0.0 (unrelated) and 1.0 (identical). Of the 13,340 pairwise comparisons, 4,670 were 0.0 (unrelated). Examples of varieties that showed a high number of zero coefficients (>110 pairwise comparisons) were Aziziah (113), Russello (115), Timilia (115), Tangarog (115), Kyperonda (115), Gianni (115) Cosmodur (115), Italo (115), Durfort (115), Provenzal (115) Sharm 5 (115), Athena (115), Tresor (115), Quadruro (115), Plinio (114), Doral (115), Parsifal (115), Nefer (115) and Virgilio (115). Several cultivars showed identical kinship, including Platani and Ciccio (0.185), Exeldur, Brindur and Neodur (0.092), Lambro and Belfuggito (0.072), and Vendetta, Simeto and Fortore (0.191).

As performed on the entire collection, the similarities between the distance matrices based on the SSRs, DArT and kinship coefficient were measured by comparing the three genetic distance matrices using the Mantel test. The highest correlation was observed between the SSR and DArT matrices (r = 0.48, p≤0.001), which indicates that each set of markers yielded highly similar estimates of genetic distances between genotypes. Lower but significant positive correlations were obtained between the DArT and pedigree matrices (r = 0.21, p≤0.001) and between the SSR and pedigree matrices (r = 0.23, p≤0.001). Of the three methods used, the pedigree analysis provided the lowest resolution.

## Discussion

The accurate description of population structure became extremely important with the advent of association genetics [63]. Indeed, when not accounted for sufficiently, population structure can lead to spurious detection of associations between markers and phenotypes of interest [64]. So, understanding the level and structure of the genetic diversity of a crop is a prerequisite for the conservation and efficient use of the available germplasm for plant breeding.

Several tools have been used to evaluate genetic diversity in domesticated and wild wheat, with those most widely used being morpho-agronomic traits [30], [65], seed storage proteins [14], [46], [66], and molecular markers [4], [9], [23]. In the present study, we combined the analysis of information observed for phenotypic traits (morphological and biochemical markers) with molecular marker (SSRs and DArTs) and pedigree data. The combination of different types of information might be very important for the effects of different evolutionary forces on the structure of the genetic diversity to be disentangled, and also to highlight the evolutionary history of crop germplasm. For instance, while molecular-marker neutrality can be assumed, morphological and biochemical traits are more likely to be affected by selection. Moreover, among molecular markers, differences in describing the diversity of a population might be associated with different mutation rates [67]–[68]. Finally, pedigree information is also an important element to assess the structure of the diversity of modern cultivars.

### Structure of the Whole Collection

The genetic diversity of the morphological traits and seed storage proteins was always lower in the cultivated durum group compared to the wild and domesticated emmer, while ssp. *turanicum*, ssp. *polonicum* and ssp. *carthlicum* were intermediate. In contrast, differences in the levels of molecular diversity for SSR and DArT markers were less strong, which suggests that selection for a well-defined phenotype was very strong during the domestication and development of durum cultivars. The ssp. *dicoccum* and ssp. *dicoccoides* were the ones that had the highest genetic variability for seed storage proteins and SSRs, which confirms that the unadapted germplasm (i.e., the wild and landrace germplasm) represents a powerful source of genes for the improvement of durum wheat [40]. Very low genetic diversity was observed in the ssp. *carthlicum* for all of the markers. This indicated the restricted genetic basis of these wheats, in agreement with Carmona *et al.* [66] and Riefolo *et al.* [14], which is probably associated to a more restricted area of geographical origin [69]. Moreover the molecular analysis showed ssp. *carthlicum* as a very distinct group from the other free-threshing tetraploid wheats, such as ssp. *durum*, ssp. *turgidum*, ssp. *turanicum* and ssp. *polonicum*. As it looks very much like common wheat (*T. aestivum* ssp. *vulgare*), ssp. *carthlicum* was initially classified as a hexaploid species [70]. A striking feature of ssp. *carthlicum* is the awned glume, so all of the spikelets show four awns. Later, due to its resistance to mildew and rust and a chromosome number with 2 n = 28, ssp. *carthlicum* was recognised as a tetraploid species [71].

When considering K = 2 using both SSRs and DArTs, the data were effective in discriminating about 95% of the *durum* genotypes from a second group that included all of the other accessions (122 out 128 durum cultivars were grouped together). Nevertheless the *durum* cultivars Belfuggito, Timilia, Ceedur, Kyperounda, Lambro and Russello were clustered with the other accessions. The presence in the second group of Kyperounda, Timilia and Russello might be explained as being derived from local landraces, while Belfuggito and Lambro have ssp. *dicoccoides* in their pedigree. This suggested that the cultivars Ceedur and Kyperounda could also be derived by hybridisation between an improved durum variety and a genotype extracted from a wild or landrace population.

According to Oliveira et al. [35] the structure of the dendrograms, obtained from SSR and DArT distances, strengthen the further taxonomic *T. turgidum* spp. subdivision proposed by MacKey [43], which classifies *durum*, *turanicum*, *turgidum* and *polonicum* as convarieties of the subspecies *T. turgidum* spp. *turgidum* (e.g., *T. turgidum* spp. *turgidum* conv. *durum*), and distinguishes the wild and domesticated emmer from the naked wheat gene pool.

Moreover, when some hexaploid wheat genotypes were added to the cluster analysis (data not shown), the spp. *carthlicum* accessions were more similar to common wheat than to ssp. *dicoccoides*, which confirms the uncertain origin of this subspecies [69]–[70], and suggests gene flow from hexaploid to tetraploid wheat [72]–[73].

### Divergence Analysis Across Subspecies during Wheat Evolution

Tetraploid wheat has undergone intensive selection for certain desirable characteristics during domestication and the subsequent breeding process, such as high and stable yields. A Fst-outlier method was used to identify which loci might be under positive selection, and therefore might be linked to regions of the genome that are responsible for the phenotypic variation present in the germplasm analysed.

We identified 211 candidate loci under positive selection based on Fst values that fall outside of the 99% confidence interval established for the distribution [57]. These loci might be directly under selection, but they are more likely to mark regions of the genome that have been selected during evolution. Among the 211 loci identified under selection, 109 markers were recently mapped on the durum wheat consensus map [54]. Some of these markers were clustered into specific regions on chromosome arms 2BL, 3BS and 4AL, where several genes/QTLs involved in the domestication of tetraploid wheat are located, such as the tenacious glumes (*Tg*) [74]–[78] and brittle rachis (*Br*) [77], [79] characteristics. The regions identified from the markers under selection, *wPt-6122* and *wPt-7004*, which were mapped on chromosome bin 2BL4 (0.50–0.89) [54], corresponded at the *Tg2* QTL reported by Peleg *et al.* [78]. Additional QTLs that affect threshability have been detected on chromosome 4AL [78]. In particular, there are two *Tg* QTLs on chromosome bin 4AL13 (0.59–0.66) that are linked at the markers *wPt6515*, *wPt7558* and *Xgwm610* [78]; these coincided with the same region as the *wPt8489*, *wPt5455*, *wPt6303*, *wPt1091* and *wPt6330* markers under selection that were mapped on the durum wheat consensus map [54]. Finally, the markers *wPt8686*, *wPt1349*, *rPt5853* and *wPt1159* that were identified in the present study as being under selection are in the same chromosome bin 3BS8 (0.78–1.00), to where the dominant gene *Br-A3* that affects the brittle rachis in tetraploid wheat was mapped by Nalam *et al*. [79].

These data suggest that the use of objective approaches to identify outliers will reveal portions of the genome that are under selection and that might represent candidates for further functional analyses to identify the loci underlying the phenotypic differences between these varieties.

### Population Structure of Durum Wheat Cultivars

The population structure of the durum accessions identified two major groups associated with the year of release of the cultivar. Moreover, the genetic diversity of morphological traits and seed storage proteins was always lower in the modern durum cultivars registered after 1990, than in the intermediate and old ones. This marked effect on diversity was not observed for molecular markers, where there was only a weak reduction. This suggests that the reduction in diversity is likely to be due to selection for few adaptive traits (i.e., glaucousness) and quality characters (i.e., glutenin and gliadin subunits) that strongly correlate with grain yield and technological properties of gluten. Similar results were observed in durum wheat in terms of yield components and yellow pigment content [65], [80]. The absence of a parallel reduction in the genetic diversity can be explained by two non-alternative hypotheses: (i) the effect of drift has not been very strong during modern breeding; and (ii) new germplasm from different gene pools has been introduced in the genetic background of the durum cultivars. Considering this first hypothesis, a possible explanation arises as the durum diversity was already significantly reduced at the very beginning of the breeding, while a second explanation might be associated with the introduction of new genotypes from CYMMIT or from countries such as France, Spain and USA. The second hypothesis is supported by data obtained by [24], and it can also explain the association between the population structure and the ‘ages’ of the different varieties.

### Correlation between Diversity and Distance Estimates

The average genetic distances between the pairs of durum accessions that were obtained for molecular markers differed markedly. With DArT markers, a low mean genetic distance was obtained (0.33; range 0.0–0.49), whereas the pedigree information yielded a mean distance of 0.89 (range 0.0–1.0). SSRs produced a range of pairwise distances between 0.00 and 0.96, with a mean of 0.63. The low distances seen between these lines using DArT markers probably reflects the limited number of alleles (presence vs. absence) compared to SSRs. Thus, for any pair of accessions, a large proportion of the markers carries the same allele, even between very different genotypes. In contrast, using SSRs, the greater number of alleles increases the probability that two genotypes differ for any given marker. At the other extreme, pedigree information leads to a highly biased distribution of genetic distances (data not shown), with a large number of pairs showing a distance of 1, thus indicating that there are no common ancestors in their pedigrees. This bias in the distribution of distances based on the pedigree information also impacts heavily on the correlation between the genetic distances.

As reported above, the correlations between pedigree *versus* the SSR and DArT matrices were significant, but low (0.23 and 0.21, respectively), although in many previous studies, even weaker correlations (0.10–0.25) have been reported between genetic distance and similarities based on pedigree and molecular markers, such as single nucleotide polymorphism [25], random amplification of polymorphic DNA [81], restriction fragment length polymorphism [82] and amplified fragment length polymorphism [83].

The low level of genome similarities obtained from parentage analysis might be due to the distinct names that are often given to parents, which actually trace back to common progenitors and incomplete or uninformative pedigree records, especially if encoded varieties are included [83]. Nineteen of 116 durum genotypes appear to be unique, with no commonality with other genotypes. In addition to this, various assumptions that are made in the calculation of kinship coefficients based on pedigree can introduce inaccuracies [13], [84]–[85]. These include: (i) the equal parental contributions to progeny; (ii) the absence of selection pressure or genetic drift; and (iii) the absence of relatedness of parents with unknown pedigree. So, the genetic similarity estimates based on molecular marker data are expected to be more accurate, as any polymorphism is a direct outcome of variations at the DNA level.

Most of the durum cultivars included in the present study were selected in Italy. The pedigree information indicated that many of these cultivars derived from a few durum lines. In particular, the Cappelli genotype can be considered as the true founder of the germplasm of the cultivated durum wheats. Moreover, Creso, Valnova and Valforte can be considered as the main founders of the modern durum cultivars. Indeed, these three genotypes, which are known as the first generation of the modern CIMMITY-related materials, are historically relevant in that they introduced the innovative semi-dwarf CIMMITY materials into the Italian durum germplasm [24]. This suggests that most of the varieties introduced and the advanced breeding lines developed by crossing exotic materials (introduced by CIMMYT, or from Mexico, USA, France), or the genotypes derived from exotic materials followed by selection of superior genotypes, make the gene pool smaller for all of the wheat cultivars. Therefore, there is the need to incorporate new variability into the existing wheat germplasm to address the new challenges, like climate change and food security.

Our data also demonstrate that dendrograms obtained with the two types of marker data are highly congruent, and that the durum cultivars were clearly divided into two major groups that reflect their origins and year of release. With few exceptions, such a clear division was also documented in a small collection of 28 durum cultivars by Maccaferri *et al.* [23] using SSR markers, and Zhang *et al.* [86] using DArT markers. This is, however, quite different from the situation reported by Rostoks *et al.* [87] among European barley cultivars, where the habitus (winter *vs.* spring) was found to be the primary determinant of the population structure. This suggests that for the most part of the last century, Italian breeders used predominantly Italian genetic material in their breeding programmes, and only in the last decades did they perform crosses using genetic material that came from other countries.

### Conclusions

In conclusion, our data initially confirm that both sets of SSR and DArT markers provide an accurate picture of the population structure within tetraploid wheat collections, which is information of critical importance for the design of association analyses. Probably a subsequent increase in the number of accessions will allow us to better understand the influence of the geographical area of origin on the evolutionary behaviour of tetraploid wheats. The present study also suggests the genetic potential of the landraces and wild accessions for the detection of unexplored alleles. Overall, the panel of genotypes investigated in the present study represents a strategic platform for the study of traits related to evolution and domestication of tetraploid subspecies, and for association mapping studies. The information obtained from this collection of genotypes will help in the selection of parents to develop high-yield durum wheat lines in breeding programmes, and to determine the potential of this panel of varieties for association mapping in subsequent studies.

## Supporting Information

Table S1 Year of release, country and pedigree information of the 128 durum wheat accessions assembled in the wheat collection.
(DOCX)

Table S2 Alleles number and genetic diversity for each SSR marker for each subspecies included in the wheat collection.
(DOCX)

Table S3 DArT markers under selection, chromosome position and putative function.
(XLSX)
