# Supplementary material for: Genetic Diversity and Population Structure of Tetraploid Wheats (Triticum turgidum L.) Estimated by SSR, DArT and Pedigree Data
Source: PLoS One. 2013 Jun 27;8(6):e67280. doi: 10.1371/journal.pone.0067280 (PMC3694930; doi:10.1371/journal.pone.0067280)
Supplement: Table S1 — Year of release, country and pedigree information of the 128 durum wheat accessions assembled in the wheat collection. (DOCX) [file pone.0067280.s001.docx]

**Table S1**. Year of release, country and pedigree information of the 128 durum wheat accessions assembled in the wheat collection.

| **Taxonomic classification** | **Year of release** | **Accession** | **Country** | **Pedigree** |
| --- | --- | --- | --- | --- |
| *T. turgidum* | 1915 | Cappelli | Italy | Strampelli selection from “Jean Retifah” |
| ssp. *durum* | 1925 | Aziziah | Italy | Selected from exotic landraces “Near East” |
|  | 1928 | Russello | Italy | Selected from landraces Italian |
|  | 1930 | Timilia | Italy, Sicily | Selected from landraces of South Italy |
|  | 1934 | Taganrog | Italy | Selected from landraces Russian |
|  | 1940 | Capeiti-8 | Italy | Eiti 6/Cappelli |
|  | 1949 | Grifoni | Italy | Cappelli/*Triticum aestivum* |
|  | 1956 | Kyperounda | Marocco | Selected from landraces |
|  | 1956 | Langdon | United States | Yuma (*T. turgidum* ssp *durum*)/Stewart (*T. turgidum* ssp. *durum*)//Carleton |
|  | 1970 | Hymera | Italy | B-14/Capeiti-8 |
|  | 1970 | Trinakria | Italy | B-14/Capeiti-8 |
|  | 1973 | Appulo | Italy | Cappelli/Grifoni//Capeiti-8 |
|  | 1973 | Belfuggito | Italy | Conte Morando/3/ *T. turgidum* ssp *dicoccoides* /Duro Australiano// *T. persicum* var *ekinatum*/ Duro Australiano |
|  | 1973 | Lambro | Italy | Conte Morando/3/ *T. turgidum* ssp *dicoccoides* /Duro Australiano// *T. persicum* var *ekinatum*/ Duro Australiano |
|  | 1974 | Creso | Italy | Yaktana-54/Norin 10-B//2*Cappelli-63/3/3*Tehuacan-60/4/Capelli-B144 |
|  | 1975 | Isa | Italy | Sassari 0 130/Patrizio |
|  | 1975 | Mexicali 75 | Mexico | Gerardo-VZ-469/3/Jori (SIB)//ND-61-130/Leeds |
|  | 1975 | Mida | Italy | Yaktana-54/Norin 10-B//2*Cappelli-63/3/2*Tehuacan-60/4/Capelli-B144 |
|  | 1975 | Polesine | Italy | Forlani/Aziziah |
|  | 1975 | Valgerardo | Italy | Giorgio-324//Senatore Cappelli/Yuma |
|  | 1975 | Valnova | Italy | Giorgio-324//Senatore Cappelli/Yuma |
|  | 1976 | Tito | Italy | Lakota/Castelporziano |
|  | 1977 | Sansone | Italy | Mara/Cappelli |
|  | 1980 | Karel | Italy | Mx-198/Maristella |
|  | 1980 | Produra | United States | Tremes-Molle-Enano/2*Tehuacan-60/3/Zenati-Bouteille/Wells/4/2*Barrigon-Yaqui-Enano/Tehuacan-60/Tacur-Tipo-125-E/2*Tehuacan-60 |
|  | 1980 | Valforte | Italy | VZ-156/Cappelli//Yuma/2*Cappelli |
|  | 1981 | Berillo | Italy | *Haynaldia* *villosa*/3*Cappelli/5/Yakatana-54//Norin-10/Brevor/3/ST-464/6/2*Thatcher |
|  | 1982 | Appio | Italy | Cappelli//Gaviota/Yuma |
|  | 1982 | Athena | Italy | Ranieri/Jucci |
|  | 1982 | Latino | Italy | Cappelli/Anhinga//*T. turgidum* |
|  | 1982 | Messapia | Italy | Mex/Crane (SIB)//Tito |
|  | 1983 | Arcangelo | Italy | Creso/Appulo |
|  | 1983 | Lloyd | United States | Cando/Edmore |
|  | 1984 | Altar84 | Mexico | Ruff/Flamingo, Mex//Mexicali 75/3/Shearwater |
|  | 1984 | Duilio | Italy | Cappelli//Anhinga/Flamingo, Mex |
|  | 1984 | Primadur | France | Blondur//2587-8-6/Leeds |
|  | 1984 | Quadruro | Italy | SC-146/ID83-NE |
|  | 1984 | Tresor | Italy | Amber durum/S-22-80 |
|  | 1985 | Adamello | Italy | Valforte/Turkis line 7112 |
|  | 1985 | Grazia | Italy | Leeds (M 6800127)/Valselva |
|  | 1986 | Ambral | France | D-76018/Valdur |
|  | 1987 | Amedeo | Italy | Maristella/Capeiti |
|  | 1987 | Brindur | France | Crosby/623//Edmore |
|  | 1987 | Neodur | France | 184-7/Valdur//Edmore |
|  | 1988 | Agridur | France | Edmore//Cimmyt 303/Chandur |
|  | 1988 | Antas | Italy | Barrigon-Yaqui-Enano/4/Tehuacan-60/3/Yaktana-54//Norin-10/Brevor/5/Tacur-Tipo-125//Tehuacan-60/Ichnusa |
|  | 1988 | Plinio | Italy | Linea D50/Trigo Candeal |
|  | 1988 | Simeto | Italy | Capeit-8/Valnova |
|  | 1990 | Fenix | Italy | Madif/Durum Tunisian//Valgerardo |
|  | 1990 | Ofanto | Italy | Adamello/Appulo |
|  | 1991 | Enduro | Italy | Gaviota/Tehuacan-60//Mexicali-75 |
|  | 1992 | Cirillo | Italy | Jucci/Polesine//Creso/Montanari |
|  | 1992 | Cosmodur | France | Natural hybridization of D881 line |
|  | 1992 | Dauno | Italy | Gediz-75/Flamingo, Mex//Teal, Mex |
|  | 1992 | Doral | France | INRA-164-1-27/IDSN-45 |
|  | 1992 | Exeldur | France | Valdur/Regal |
|  | 1992 | Fauno | Italy | Gediz-75/Flamingo, Mex//Teal, Mex |
|  | 1992 | Gianni | Italy | Multiple cross among durum wheat cultivar |
|  | 1992 | Granizo | Spain | Yel S/Shaw S |
|  | 1992 | Parsifal | France | INRA 92-1/D81028 |
|  | 1992 | Zenit | Italy | Valriccardo/Vic |
|  | 1993 | Italo | Italy | Complex cross between Italian and Turkish genotypes |
|  | 1993 | Kronos | United States | APB MSFRS POP Sel (D03-12) |
|  | 1994 | Ceedur | France | Mondur/2587.8.6//Edmore/Chandur |
|  | 1995 | Arcobaleno | Italy/Spain | Chen/Altar 84 |
|  | 1995 | Ares | Italy | Lira/Vic |
|  | 1995 | Colosseo | Italy | Mutant Mexa/Creso |
|  | 1995 | Fortore | Italy | Capeiti-8/Valforte |
|  | 1995 | Platani | Italy | Valnova/Capeiti-8 |
|  | 1995 | Preco | Italy | Edmore/WPB881//Selected line 3 |
|  | 1995 | Saadi | France | IDSM72-3/711.8 |
|  | 1996 | Bronte | Italy | Berillo/Latino |
|  | 1996 | Ciccio | Italy | F6 Appulo/Valnova//F5 Valforte/Patrizio |
|  | 1996 | Durfort | France | Selected from REVA population |
|  | 1996 | Iride | Italy | Altar 84/Ares |
|  | 1996 | Nefer | France | 164/Keops |
|  | 1996 | Rusticano | Italy | n.a. |
|  | 1996 | San Carlo | Italy | Grazia/Degamit |
|  | 1996 | Svevo | Italy | CIMMYT's selection/Zenit |
|  | 1996 | Vitromax | Italy/Spain | Turchia77/3/Jori/Anhunga//Flamingo, Mex |
|  | 1997 | Varano | Italy | Capeiti-8/Creso//Creso/3/Valforte/Trinakria |
|  | 1998 | AC Navigator | Canada | Kyle/WB 881 |
|  | 1998 | Baio | Italy | Duilio/F21//G76 |
|  | 1998 | Cannizzo | Italy | F5 Capeiti/Valnova// F5 Patrizio/Valforte |
|  | 1998 | Claudio | Italy | CIMMYT's selection 35/Durango//IS1938/Grazia |
|  | 1998 | Martino | Italy | Appulo/Produra |
|  | 1998 | Provenzal | Italy | CIMMYT's selection (ATO’S CII3EIPUSOA 580) |
|  | 1999 | Giotto | Italy | W.A 6518-2/GA547 |
|  | 1999 | Meridiano | Italy | Simeto/WB 881/Duilio/F21 |
|  | 1999 | Orobel | Italy | Composite INRA/Gil Ble Dur |
|  | 1999 | Quadrato | Italy | Creso/Trinakria |
|  | 1999 | Vesuvio | Italy | Ofanto/Simeto |
|  | 2001 | Avispa | Italy | n.a. |
|  | 2001 | Fiore | Italy | Derived from CIMMYT's selection |
|  | 2001 | Tiziana | Italy | Peleo/Neodur |
|  | 2002 | Duetto | Italy | 1485 x 83.74 |
|  | 2002 | Dylan | Italy | Neodur/Ulisse |
|  | 2002 | Grecale | Italy | S2/WB 881//Plinio/F22 |
|  | 2002 | Normanno | Italy | Simeto/F22//L35 |
|  | 2002 | Virgilio | France | Acalou/Shoula |
|  | 2003 | Ancomarzio | Italy | Stotka//Altar84/Ald |
|  | 2003 | Casanova | Italy | Flavio/Syene//Duilio |
|  | 2003 | Chiara | Italy | Arcangelo/Fortore |
|  | 2003 | Latinur | France | n.a. |
|  | 2003 | Vendetta | Italy | Creso/Ofanto |
|  | 2004 | L092 | United States | 1A. 1D/Len//Langdon/3/2*Renville |
|  | 2004 | L252 | United States | 1A. 1D/Len//Langdon/3/2*Renville |
|  | 2004 | Maestrale | Italy | Iride/Svevo |
|  | 2004 | Orfeo | Italy | Creso/Simeto |
|  | 2004 | S99B34 | United States | 1A. 1D/Len//Langdon/3/2*Renville |
|  | 2004 | Saragolla | Italy | Iride/Line SPB 0114 |
|  | 2005 | Ariosto | Italy | Karim/GA7-X3//Duilio |
|  | 2005 | Arnacoris | Italy | n.a. |
|  | 2005 | Canyon | Italy | n.a. |
|  | 2005 | Imhotep | Italy | n.a. |
|  | 2005 | PR22D89 | Italy | Ofanto/Duilio//Ixos |
|  | 2005 | Strongfield | Canada | AC-Avonlea/DT-665 |
|  | 2006 | Alemanno | Italy | Cappelli/Duilio |
|  | 2006 | Ciclope | Italy | Trinakria/Berillo//Valnova/Trinakria |
|  | 2006 | K26 | Italy | Spontaneuos mutant derived from Simeto/Colosseo |
|  | 2006 | UC1113 | Canada | [Kingfisher //Rossia/BD-1419/3/Mexi/CP/4/Waha/5/Yavaros-79](http://genbank.vurv.cz/wheat/pedigree/krizeni3.asp?id=%2773869%27) |
|  | 2007 | Neolatino | Italy | Latino/Trinakria//MG1433/4/Latino |
|  | - | 5-BIL42 | Italy | Breeding line derived from Latino/MG29896 |
|  | - | PC32 | Italy | Breeding line derived from F6 Latino/Primadur |
|  | n.a. | Barcarol | Italy | n.a. |
|  | n.a. | Pedroso | Spain | n.a. |
|  | n.a. | Sharm 5 | Syria | Selected from landraces Syrian |
|  | n.a. | West Bread 881 | United States | Complex cross of Ward, Wells, Cando, Waskana, Mexicali 75 |

n.a. not available
