# Supplementary material for: Genetic Diversity and Population Structure of Tetraploid Wheats (Triticum turgidum L.) Estimated by SSR, DArT and Pedigree Data
Source: PLoS One. 2013 Jun 27;8(6):e67280. doi: 10.1371/journal.pone.0067280 (PMC3694930; doi:10.1371/journal.pone.0067280)
Supplement: Table S2 — Alleles number and genetic diversity for each SSR marker for each subspecies included in the wheat collection. (DOCX) [file pone.0067280.s002.docx]

**Table S2**. Alleles number and genetic diversity for each SSR marker for each subspecies included in the wheat collection.

|  | Chromosome arms | *T. turgidum* | | | | | | |
| --- | --- | --- | --- | --- | --- | --- | --- | --- |
|  |  | ssp. *durum* | ssp. *turanicum* | ssp. *polonicum* | ssp. *turgidum* | ssp. *carthlicum* | ssp. *dicoccum* | ssp. *dicoccoides* |
| Sample size |  | 128 | 20 | 20 | 19 | 12 | 18 | 12 |
| *Xcfd15* | 1AS | 9 | 8 | 8 | 10 | 5 | 11 | 6 |
| *Xgwm633* | 1AL | 5 | 3 | 2 | 5 | 4 | 5 | 7 |
| *Xwmc406* | 1BS | 5 | 4 | 6 | 4 | 8 | 6 | 6 |
| *Xgwm124* | 1BL | 5 | 2 | 2 | 2 | 3 | 3 | 2 |
| *Xgwm311* | 2AL | 8 | 8 | 6 | 5 | 6 | 9 | 6 |
| *BQ170801^*^* | 2BS | 2 | 4 | 4 | 2 | 1 | 4 | 4 |
| *Xbarc45* | 3AS | 6 | 5 | 5 | 4 | 4 | 6 | 4 |
| *Xgwm1042* | 3AL | 7 | 4 | 5 | 5 | 5 | 7 | 5 |
| *BJ274952^*^* | 3BS | 2 | 3 | 2 | 2 | 1 | 3 | 2 |
| *Xgwm299* | 3BL | 8 | 8 | 5 | 9 | 4 | 4 | 7 |
| *Xgwm1093* | 4AS | 14 | 8 | 9 | 7 | 8 | 12 | 7 |
| *Xgwm937* | 4AL | 11 | 7 | 7 | 9 | 5 | 6 | 6 |
| *Xgwm1084* | 4BS | 10 | 3 | 5 | 9 | 6 | 9 | 7 |
| *Xgwm495* | 4BL | 8 | 5 | 6 | 5 | 5 | 6 | 5 |
| *Xgwm154* | 5AS | 5 | 4 | 5 | 5 | 6 | 5 | 7 |
| *Xgwm865* | 5AL | 13 | 6 | 7 | 8 | 7 | 8 | 6 |
| *Xgwm408* | 5BL | 6 | 4 | 3 | 5 | 3 | 6 | 8 |
| *Xgwm499* | 5BL | 10 | 7 | 7 | 7 | 3 | 7 | 6 |
| *Xwmc235* | 5BL | 10 | 8 | 5 | 5 | 6 | 7 | 5 |
| *Xgwm1017* | 6AL | 9 | 5 | 6 | 8 | 6 | 6 | 5 |
| *Xgwm459* | 6BS | 14 | 11 | 6 | 9 | 8 | 9 | 7 |
| *Xgwm193* | 6BS | 5 | 4 | 4 | 4 | 4 | 5 | 7 |
| *Xgwm60* | 7AS | 6 | 4 | 4 | 7 | 4 | 7 | 5 |
| *Xgwm1066* | 7AL | 6 | 4 | 6 | 4 | 4 | 5 | 7 |
| *Xgwm537* | 7BS | 9 | 6 | 5 | 7 | 8 | 8 | 8 |
| *Xwmc606* | 7BS | 18 | 10 | 10 | 11 | 9 | 13 | 6 |
| n_a_ total |  | 211 | 145 | 140 | 158 | 133 | 177 | 151 |
| H_E_ mean |  | 0.60 | 0.58 | 0.58 | 0.62 | 0.56 | 0.66 | 0.70 |

* EST-SSR markers; *n_a_*: number of alleles; H_E_: genetic diversity.
